# Tai Chi Exercise Increases SOD Activity and Total Antioxidant Status in Saliva and Is Linked to an Improvement of Periodontal Disease in the Elderly

**DOI:** 10.1155/2014/603853

**Published:** 2014-03-26

**Authors:** Víctor Manuel Mendoza-Núñez, Beatriz Hernández-Monjaraz, Edelmiro Santiago-Osorio, José Miguel Betancourt-Rule, Mirna Ruiz-Ramos

**Affiliations:** ^1^Unidad de Investigación en Gerontología, Facultad de Estudios Superiores Zaragoza, Universidad Nacional Autónoma de México (UNAM), Guelatao No. 66, Delegación Iztapalapa, 09230 México, DF, Mexico; ^2^Laboratorio de Biología Celular y Molecular del Cáncer, UIDCC, FES-Zaragoza, UNAM, México, DF, Mexico; ^3^Departamento de Ciencias de la Salud, Universidad Autónoma Metropolitana-Iztapalapa, Apartado Postal 55-535, 09340 México, DF, Mexico

## Abstract

The aim of this study was to determine the effect of Tai Chi on biological markers of oxidative stress in saliva and its relationship with periodontal disease (PD) in older adults. We carried out a quasi-experimental study with a sample of 71 sedentary volunteers with PD who were divided into a control group of 34 subjects and an experimental group of 37 subjects who performed Tai Chi 5 days a week for a period of 6 months. PD status was characterized using the Periodontal Disease Index (PDI). Superoxide dismutase (SOD), total antioxidant status (TAS), and TBARS levels of both groups were measured by spectrophotometric methods. In addition, inflammation markers (TNF-**α**, IL-1**β**, IL-6, IL-8, and IL-10) were measured by flow cytometry. We found a statistically significant increase in SOD activity (*P* < 0.001) and TAS concentration (*P* < 0.05), whereas levels of IL-1**β** were significantly lower (*P* < 0.01). Likewise, a statistically significant decrease in the PDI (*P* < 0.05) was observed in subjects who performed Tai Chi during a period of 6 months. Our findings suggest that the practice of Tai Chi has both antioxidant and anti-inflammatory effects that are linked to the improvement of PD in older adults.

## 1. Introduction

Tai Chi (TC) is a traditional Chinese exercise linked to martial arts that has been shown to have a positive effect on aerobic capacity, muscle strength, balance, and motor control [1]. The practice of TC involves exercises that promote posture, flexibility, relaxation, wellness, and mental concentration [2]. TC is characterized by extremely slow movements, absolute continuity without interruption or pause, and a total awareness and focus on its implementation [3]. Unlike many exercises that are characterized by muscle strength and effort, TC movements are slow, soft, and lightweight [1–3]. TC is classified as a moderate type of exercise, as its intensity does not exceed 55% of an individual's maximum oxygen expenditure and 60% of an individual's maximum heart rate [4].

Recently, the practice of TC has been promoted in the elderly population due to its beneficial health effects, including, among others, the prevention of falls, osteoporosis, hypertension, and diabetes mellitus as well as rheumatological and neurological disorders [2, 5, 6]. Our research group has shown that regular practice of TC increases superoxide dismutase activity and total antioxidant status in the serum of the elderly [7, 8]. We hypothesize that the antioxidant effect of TC may also be observed in saliva and could have a positive effect on the oral health of the elderly. Periodontal disease (PD) is one of the major chronic oral diseases in the elderly and is characterized by a destructive inflammatory process that affects the supporting tissues of the teeth, causing both alveolar bone resorption and formation of periodontal pockets and eventually leads to tooth loss [9, 10]. It has been demonstrated that oxidative stress in the saliva is an etiologic factor and pathophysiologic of PD [11–15]. Therefore, the aim of this study is to determine the effect of Tai Chi on biological markers of oxidative stress in the saliva as well as its relationship with periodontal disease in the elderly.

## 2. Methods 

### 2.1. Design and Subjects

A quasi-experimental study was performed with a sample size of 71 sedentary volunteers with a clinical diagnostic of periodontal disease. The age range of the subjects was 60–74 years. Volunteers taking nutritional supplements or anti-inflammatory medications were excluded from the study (*n* = 10). All participants gave their written, informed consent for inclusion in the study. The investigation protocol was approved by the Ethics Committee of the Universidad Nacional Autónoma de México (UNAM), Zaragoza Campus (IN306213-2).

**Figure 1:**
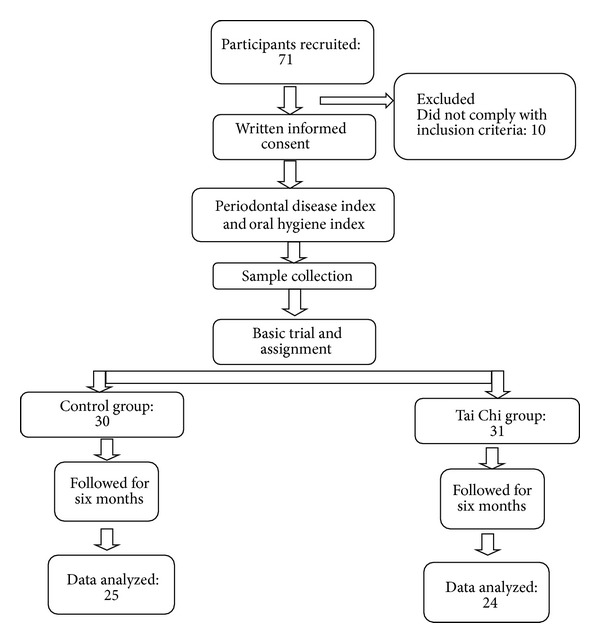
General scheme for study tracking.

Subjects were divided into two groups: a control group (CG) with 30 subjects who did not exercise and an experimental group (EG) with 31 subjects who performed Tai Chi (Eight-Form) [16] 5 days a week for 60-minute sessions under the supervision of a qualified instructor for 6 months (Figure 1). Twelve subjects (5 from the experimental and 7 from the control group) were excluded from the study analysis as they were unable to complete the study.

### 2.2. Periodontal Health Status

The periodontal health status of each subject was measured using the Periodontal Disease Index (PDI). The examination procedure involved the insertion of a graduated periodontal probe between the subjects' teeth and gums at a standard force to measure pocket depth. These assessments were made for sextants of the dentition, with the third molars only included if the second molars were missing. The final periodontal disease score was determined by taking the mean of the sextant scores [17].

Oral cleanliness was measured using the Oral Hygiene Index-Simplified (OHI-S). Oral debris and calculus were estimated by running the side of an explorer along the surface of the examined teeth, including the upper first molars (teeth 16 and 26), the lingual faces of the lower first molars (teeth 36 and 46), and the labial faces of the upper right (tooth 11) and lower left (tooth 31) incisors [18].

### 2.3. Sample Collection and Preparation

Whole unstimulated saliva samples were collected from both groups (control and experimental) before (baseline) and after the six-month period. The samples were obtained one to two hours after an eight-hour fasting period and were collected in 15 mL polypropylene tubes. Saliva was allowed to pool in the bottom of the mouth and was drained into the collection tube. At the end of the collection period, saliva samples were centrifuged at 2,500 rpm for 10 minutes. The supernatant fraction was then aliquoted into storage vials and kept at −80°C until further analysis.

### 2.4. Saliva TBARS

The TBARS assay was performed using whole saliva, as described by Jentzsch et al. (1996) [19]. In the TBARS assay, one molecule of malondialdehyde reacts with two molecules of thiobarbituric acid (TBA), producing a pink pigment with an absorption peak of 535 nm. Amplification of peroxidation during the assay is prevented by the addition of the chain-breaking antioxidant butylated hydroxytoluene (BHT).

### 2.5. Saliva Total Antioxidant Status (TAS)

Antioxidant quantification was performed by monitoring 2,2′-azino-bis(3-ethylbenzthiazoline-6-sulfonic acid) (ABTS+) radical formation (Randox Laboratories, Ltd., Crumlin Co., UK). The antioxidants present suppressed the bluish-green staining of the ABTS+ cation, which is proportional to the antioxidant concentration level. The reaction kinetics were measured using a colorimetric technique in an Autoanalyzer Vitalab Eclipse Merck (Dieren, The Netherlands) [20].

### 2.6. Saliva Superoxide Dismutase (SOD)

Xanthine and xanthine oxidase (XOD) were used to generate superoxide radicals, which react with 2-(4-iodophenyl)-3-(4-nitrophenyl)-5-phenyltetrazolium chloride to produce a red formazan dye. SOD activity was assessed by measuring the degree of inhibition of the reaction (Randox Laboratories Ltd., Crumlin Co., UK). The kinetics of SOD activity were measured using a colorimetric technique in an Autoanalyzer Vitalab Eclipse Merck (Dieren, The Netherlands) [21].

### 2.7. Quantification of Cytokines

Aliquots of each saliva sample were assayed by flow cytometry (CBA Kit, Human Inflammatory Cytokine, BD Biosciences, Becton, Dickinson and Company, USA) to determine the levels of interleukin 1-beta (IL-1*β*), interleukin 6 (IL6), interleukin 8 (IL-8), interleukin 10 (IL-10), and tumor necrosis factor-alpha (TNF-*α*) [22].

### 2.8. Statistical Analysis

Data were analyzed using descriptive statistics, where we determined the mean and standard error (SE) and performed a repeated measures analysis of variance (repeated measures ANOVA). A *P* value of <0.05 was considered statistically significant. *P* values were determined using the statistical analysis program SPSS, version 16.0.

## 3. Results

### 3.1. Biochemical Characteristics

In Table 1, the biochemical values related to glycosylated hemoglobin, cholesterol, triglycerides, HDL, and Albumin for baseline and six months later are shown, revealing no statistically significant differences between the groups.

### 3.2. Changes in Oxidative Stress Markers by Intervention

With respect to the oxidative stress markers in saliva, a significant increase in the total antioxidant activity (0.53 ± 0.33 mmol/L at baseline versus 0.70 ± 0.35 mmol/L after intervention, *P* < 0.01) and in SOD activity (1.62 ± 0.83 UI/L at baseline versus 2.79 ± 1.6 UI/L after intervention, *P* < 0.001) was found in the group that practiced Tai Chi for six months. No significant differences were observed in the values of lipoperoxides (*P* > 0.05) (Table 2).

### 3.3. Changes in Inflammatory Markers by Intervention

A significant decrease in the concentration of interleukin 1*β* in saliva (783.62 ± 174.9 pg/mL at baseline versus 624.97 ± 196.7 pg/mL after intervention, *P* < 0.01) was found in the experimental group after practicing Tai Chi. Additionally, a borderline statistically significant decrease in the concentration of IL-6 was observed in the same group (18.66 ± 7.25 pg/mL at baseline versus 4.76 ± 1.93 pg/mL after intervention, *P* = 0.09) (Table 3).

### 3.4. Changes in Periodontal Disease by Intervention

No significant differences were observed in the oral hygiene index-simplified in any of the groups (*P* > 0.05). However, a statistically significant decrease in the periodontal disease index was observed in the experimental group (3.62 ± 0.9 baseline versus 3.28 ± 0.8 after intervention, *P* < 0.05) (Table 4).

## 4. Discussion

Oxidative stress (OxS) and chronic inflammation (CI) are biological changes inherent to aging and are risk factors for several chronic degenerative diseases such as periodontal disease (PD), one of the most prevalent aging-related diseases [23–25]. Several preventive and therapeutic options have been proposed to counteract these biochemical alterations, including, among others, dietary supplementation resulting in antioxidant effects, antioxidant vitamins, and administration of NSAIDs at low doses [26–29]. It has also recently been shown that a healthy diet and regular moderate physical exercise have antioxidant and anti-inflammatory effects, reducing the risk of chronic diseases or contributing to its treatment [30–32]. Walking and the practice of TC are among the modalities of moderate physical exercise recommended for the maintenance or improvement of health in the elderly [1, 33]. Some studies have shown that Tai Chi has positive effects on cardiorespiratory function and the musculoskeletal system, improving a person's ability to control their posture and balance which, consequently, decreases their frequency of falls [1–3]. Likewise, it has been shown that the practice of Tai Chi has a positive effect on the efficiency of the antioxidant system in adult subjects and the elderly. Thus, it has been proposed that the practice of Tai Chi could prevent and control chronic, degenerative diseases that occur with age [7, 8, 34, 35]. Additionally, it has been observed that the regular practice of physical exercise has been linked to a significantly lower frequency of periodontal disease in adult subjects [36–39].

Our results indicate a statistically significant decrease in the rate of periodontal disease in subjects practicing Tai Chi. These findings support the proposal that regular physical exercise promotes biological changes that positively impact the pathophysiological process of periodontal disease. A significant increase in the total antioxidant status and SOD activity in the experimental group were found, suggesting that one of the possible mechanisms involved in the improvement of periodontal disease is the antioxidant effect brought about by the practice of Tai Chi. Oxidative stress has been linked to the pathophysiology of PD, because reactive oxygen species (ROS) may selectively damage proteoglycans associated with soft periodontal tissues and the alveolar bone as well as chains of proline type 1 collagen, significantly altering fibroblast functions such as adhesion and proliferation as well as their half-life [40–43]. The excessive production of ROS by neutrophils and fibroblasts in periodontal tissues activates NF-*κ*B and triggers the signaling cascade that activates osteoclasts, leading to inflammation [44]. Additionally, the generation of OxS causes an imbalance of metalloproteinases and their tissue inhibitors, leading to the degradation of periodontal tissue [45].

It has also been shown that physical exercise lowers the levels of markers of inflammation [30, 46, 47]. Thus, the decrease of IL-1*β* observed in saliva in the experimental group suggests that the practice of Tai Chi has an anti-inflammatory effect on periodontal tissue. The mechanisms of these changes have not been fully elucidated, but it has been proposed that this effect may result from the regulation of cytokine expression caused by muscle contractions during exercise [48].

Finally, it has been noted that the effect of moderate physical exercise, such as Tai Chi, on oxidative stress is linked to an adaptive process influenced by the change in the body's redox balance in favor of more alkaline conditions in the cell. The reactive species generated during physical activity act as the signal that is necessary for the activation of the MAPK proteins p38 and ERK1/ERK2, which, in turn, activate the transcription factor sensitive to the redox state, NF-*κ*B, via activation of the kinase that phosphorylates the inhibitor of this factor (I*κ*B). Once freed of its inhibitor, NF-*κ*B is transported into the nucleus where it can promote the synthesis of various antioxidant enzymes, such as MnSOD [7, 8, 45, 49, 50]. Our findings suggest that the antioxidant effect of Tai Chi has a beneficial effect on oral health in the elderly.

## 5. Conclusions

Our results show that the practice of Tai Chi has an antioxidant and anti-inflammatory oral effect, supporting the proposal that the regular practice this type of exercise can contribute to the prevention and control of periodontal disease during the aging process. Thus, our findings support the proposal to recommend the regular practice of Tai Chi as a coadjuvant for the prevention and treatment of periodontal disease in older adults.

## Figures and Tables

**Table 1:** Biochemical parameters of the study population by group.

	Control (*n* = 25)		Tai Chi (n = 24)	
	Baseline	Six months	Baseline	Six months
HbA1c (%)	6.56 ± 1.80	8.96 ± 2.4	8.09 ± 2.2	7.29 ± 2.3
Cholesterol (mg/dL)	213.3 ± 39.2	209.6 ± 29.4	212.6 ± 44.2	209.5 ± 36.6
Triglycerides (mg/dL)	175.1 ± 38.5	207.6 ± 41.9	160.8 ± 76.4	195.8 ± 130
HDL (mg/dL)	39.33 ± 8.02	40.33 ± 3.5	44.375 ± 9.6	52.70 ± 11.98
Albumin (g/dL)	4.76 ± 0.05	4.77 ± 0.12	4.75 ± 0.20	4.69 ± 0.25

Values are means ± SE. ANOVA *P* > 0.05. HbA1c: glycosylated hemoglobin; HDL: High-density lipoproteins.

**Table 2:** Oxidative stress markers, baseline, and postintervention by group.

	Control (*n* = 25)		Tai Chi (n = 24)	
	Baseline	Six months	Baseline	Six months
TAS (mmol/L)	0.72 ± 0.35	0.62 ± 0.29	0.53 ± 0.33	0.70 ± 0.35*
SOD (UI/L)	2.63 ± 1.8	2.33 ± 1.1	1.62 ± 0.83	2.79 ± 1.6^†^
Lipoperoxides (*μ*mol/L)	0.14 ± 0.14	0.08 ± 0.09	0.11 ± 0.07	0.14 ± 0.09

Values are means ± SE. Repeated measures analysis of variance *P < 0.01; ^†^ *P* < 0.001. TAS: total antioxidant status, SOD: superoxide dismutase.

**Table 3:** Inflammatory markers, baseline, and postintervention by group.

	Control (n = 25)		Tai Chi (*n* = 24)	
	Baseline	Six months	Baseline	Six months
TNF-*α* (pg/mL)	2.004 ± 1.50	5.325 ± 2.23	0.5119 ± 0.009	4.2410 ± 0.435
IL-1*β* (pg/mL)	1180.18 ± 244	1353.37 ± 176	783.62 ± 174.9	624.97 ± 196.7*
IL-6 (pg/mL)	20.80 ± 5.01	59.45 ± 13.8	18.66 ± 7.25	4.76 ± 1.93^†^
IL-8 (pg/mL)	3560.53 ± 809	3215.66 ± 260	4971.24 ± 835	2252.42 ± 330
IL-10 (pg/mL)	3.15 ± 0.66	0.25 ± 0.21	0.21 ± 2.5	2.9 ± 1.5

Values are means ± SE. Repeated measures analysis of variance *P < 0.01; ^†^ *P* = 0.09. TNF-*α*: tumor necrosis factor alpha; IL-1*β*: interleukin 1*β*; IL-6: interleukin 6; IL-8: interleukin 8; IL-10: interleukin 10.

**Table 4:** Oral hygiene index-simplified and periodontal disease index by group.

	Control (n = 25)		Tai Chi (*n* = 24)	
	Baseline	Six months	Baseline	Six months
OHIS	2.43 ± 0.3	2.45 ± 0.2	2.45 ± 0.3	2.46 ± 0.3
PDI (mm)	3.2188 ± 0.6	3.7960 ± 0.4	3.6267 ± 0.9	3.2813 ± 0.8*

Values are means ± SE. Repeated measures analysis of variance *P < 0.05; OHIS: oral hygiene index-simplified; PDI: periodontal disease index.
